# LAPAROSCOPIC TREATMENT OF CELIAC AXIS COMPRESSION SYNDROME: CASE REPORT

**DOI:** 10.1590/S0102-6720201500030020

**Published:** 2015

**Authors:** Júlio Cezar Uili COELHO, Jean Carlos da SILVA, Micheli Fortunato DOMINGOS, João Augusto Nocera PAULIN, Guilherme Figueiró FERRONATO

**Affiliations:** Nossa Senhora das Graças Hospital, Curitiba, Paraná, Brazil

## INTRODUCTION

Celiac axis compression syndrome, also known as median arcuate ligament syndrome or Dunbar syndrome, is a rare condition. This syndrome was first reported by Harjola in 1963^6^. Dunbar described it as a clinical syndrome in his memorial paper in 1965^4^. It is characterized by compression of the celiac axis by the median arcuate ligament of the diaphragm. 

The median arcuate ligament is a fibrous arch formed at the base of the diaphragm at the level of the 12^th^ thoracic vertebra, where the left and right diaphragmatic crura join^1^. This fibrous arch forms the anterior aspect of the aortic hiatus, through which the aorta, thoracic duct, and azygos vein pass. The median arcuate ligament usually comes into contact with the aorta above the origin of the celiac axis. However, in some individuals, the it may be abnormally low and passes in front of the celiac axis, causing its compression, which is named median arcuate ligament syndrome^5^. 

Some patients with this syndrome refer severe clinical manifestations such as postprandial abdominal pain, weight loss, and vomiting. The primary treatment modality for this condition is surgical division of its fibers. The traditional surgical approach has been through an upper abdominal laparotomy incision. Roayaie et al. in 2000 reported the first patient with celiac axis compression syndrome treated by laparoscopy access. Afterwards, several authors have demonstrated that the laparoscopic access may be employed with success to treat this condition^8^. To best of our knowledge, this is the first report of laparoscopic treatment of the celiac axis compression syndrome in Brazil.

## CASE REPORT

**FIGURE 1:**
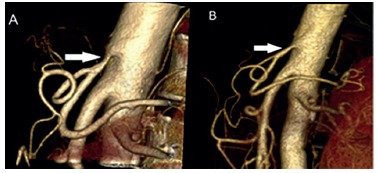
- 3D reconstruction of abdominal aortic angiotomography showing severe stenosis of the proximal segment of the celiac axis caused by extrinsic compression of the median arcuate ligament (Figure 1A, arrow). The stenosis was successfully treated by laparoscopic section of the median arcuate ligament and celiac ganglionectomy (Figure 1B, arrow).

A 60-year-old woman presented with a three-year history of intermittent postprandial epigastric pain, and weight loss of 6 kg. The abdominal pain was relieved with fasting. She denied nausea, vomiting and diarrhea. Physical examination was normal. Several exams, including abdominal ultrasonography, upper gastrointestinal endoscopy, colonoscopy, small bowel radiographic study, tomography failed to reveal any abnormality. Finally, an angiotomography showed high-grade stenosis of the anterior wall of the proximal celiac axis caused by extrinsic compression of the median arcuate ligament (Figure 1A).

The patient underwent laparoscopic section of the ligament and celiac ganglionectomy. The patient was placed in reverse Trendelenburg position with the legs abducted and supported on cushioned spreader bars. The operation was performed through five trocars inserted in the upper abdomen, similar to that of Nissen-Rosetti procedure. A right subcostal retractor was used to retract the left lobe of the liver laterally and the stomach was retracted to the patient's left side with a Babcock clamp. After dividing the gastrohepatic omentum and identifying the right crus of the diaphragm inferiorly to the cardia, the junction of both crus was carefully separated to expose the anterior surface of the aorta and identify the median arcuate ligament and celiac plexus. The median arcuate ligament that was compressing the proximal celiac axis was sectioned and all neural tissue overlying the celiac axis was resected. The operation was uneventful and lasted 70 min.

The patient was discharged from the hospital 12 h after the operation completion and had an uneventful recovery. At two-month follow-up, she referred only two episodes of mild abdominal pain and gained 3 kg. An angiotomography obtained at that time showed no celiac axis stenosis (Figure 1B).

## DISCUSSION

Since the first report of the celiac axis compression syndrome several decades ago, controversy still remains regarding the pathophysiology and clinical implications of this condition. The observation of celiac axis compression in asymptomatic patients leads to questions about the real existence of the syndrome. Some authors suggested that the clinical manifestations are caused by ischemia secondary to the reduction of blood flow through the stenotic celiac axis^2^^,^ 3 ^,^ 7. However, others claimed that pain originates from direct compression of celiac ganglia^5^^,^ 8. 

In the past, celiac axis compression syndrome was diagnosed by conventional angiography^5^. Lateral projection of aortography was the first choice to identify the celiac axis stricture. Nowadays thin-section multidetector CT scanners, associated with three-dimensional reconstruction, have become the best method to obtain high-resolution images of the aorta and its branches. Angiotomography, especially during expiration, has a high precision to identify celiac axis compression syndrome^8^. In addition, this method also allows visualization not only of the stenosed vessel but also the underlying median arcuate ligament and adherent tissue using three-dimensional imaging. Angiotomography is also important to exclude the presence of celiac axis calcifications, an important cause of arterial stricture. 

The angiotomography of this patient showed a severe stricture of the celiac axis caused by extrinsic compression of the median arcuate ligament. The stricture was successfully treated by laparoscopic section of the median arcuate ligament. Postoperative angiotomography demonstrated absence of residual stenosis of the celiac axis after the operation.

The available evidence demonstrates that both laparoscopic and open ligament release associated with celiac ganglionectomy are effective in provide celiac artery revascularization and sustained symptom relief in the majority of patients with the syndrome^2^^,^ 3 ^,^ 5. The laparoscopic approach is feasible, safe, and successful, if performed by experienced laparoscopic surgeons.

Although the laparoscopic treatment of celiac axis compression syndrome is a new technique, several authors have demonstrated its affectivity in providing symptom relief in patients^1^^,^ 2 ^,^ 8. In addition, this access has several advantages, such as reduction of postoperative pain and blood loss, shorter hospital stay and faster recovery. 

More recently, this syndrome has been effectively treated with robot-assisted surgery^3^. The advantages of this approach compared to the laparoscopic access have not yet been completed evaluated. The high cost of robot-assisted surgery is an important drawback in our country.

## References

[1] Berard X, Cau J, Déglise S, Trombert D, Saint-Lebes B, Midy D, Corpataux JM, Ricco JB (2012). Laparoscopic surgery for coeliac artery compression syndrome: current
management and technical aspects. Eur J Vasc Endovasc Surg.

[2] di Libero L, Varricchio A, Tartaglia E, Iazzetta I, Tartaglia A, Bernardo A, Bernardo R, Triscino G, Conte DL (2013). Laparoscopic treatment of celiac axis compression syndrome (CACS) and
hiatal hernia: Case report with bleeding complications and review. Int J Surg Case Rep.

[3] Do MV, Smith TA, Bazan HA, Stembergh III WC, Abbas AE, Richardson WD (2013). Laparoscopic versus robot-assisted surgery for median arcuate ligament
syndrome. Surg Endosc.

[4] Dunbar JD, Molnar W, Beman FF, Marable SA (1965). Compression of the celiac trunk and abdominal angina. Am J Roentgenol Radium Ther Nucl Med.

[5] França LHG, Mottin C (2013). Surgical treatment of Dunbar syndrome. J Vasc Bras.

[6] Harjola PT (1963). A rare obstruction of the coeliac artery: report of a
case. Ann Chir Gynaecol Fenn.

[7] Palmer OP, Tedesco M, Casey K, Lee JT, Poultsides GA (2012). Hybrid Treatment of Celiac Artery Compression (Median Arcuate
Ligament) Syndrome. Dig Dis Sci.

[8] Roayaie S, Jossart G, Gitlitz D, Lamparello P, Hollier L, Gagner M (2000). Laparoscopic release of celiac artery compression syndrome facilitated
by laparoscopic ultrasound scanning to confirm restoration of flow. J Vasc Surg.

